# Mutagenesis and characterization of a *Bacillus amyloliquefaciens* strain for *Cinnamomum camphora* seed kernel oil extraction by aqueous enzymatic method

**DOI:** 10.1186/s13568-017-0451-9

**Published:** 2017-07-17

**Authors:** Cheng Zeng, Rongbin Zhao, Maomao Ma, Zheling Zeng, Deming Gong

**Affiliations:** 1 State Key Laboratory of Food Science and Technology, Nanchang University, Nanchang, 330047 China; 2 The First School of Clinical Medicine, Nanchang University, Nanchang, 330031 China; 3 Jiangxi Province Key Laboratory of Edible and Medicinal Plant Resources, Nanchang University, Nanchang, 330031 China; 4 School of Resources, Environmental and Chemical Engineering, Nanchang University, Nanchang, 330031 China; 5 School of Food Science and Technology, Nanchang University, Nanchang, 330031 China; 6 New Zealand Institute of Natural Medicine Research, 8 Ha Crescent, Auckland, 2104 New Zealand

**Keywords:** *Bacillus amyloliquefacien*, UV and NTG mutation, Proteinase, Enzymatic properties, Aqueous enzymatic method

## Abstract

The purpose of the present study was to increase the proteinase activity of the strain NCU116 by combining ultraviolet irradiation and *N*-methyl-*N′*-nitro-*N*-nitroso guanidine treatment, in order to enhance the efficiency of *Cinnamomum camphora* seed kernel oil (CCSKO) extraction by aqueous enzymatic method (AEM). The mutated strain, designated as NCU116-1, was screened out by the ratio of hydrolytic zone diameter to colony diameter on skim milk plate. The proteinase activity (9116.1 U/ml) of NCU116-1 was increased by 31.9% compared with the parental strain. The extracellular enzymes produced by NCU116-1 included proteinase, pectase, glucoamylase, cellulase and amylase. The proteinase had the maximum activity at 50 °C. Its optimum temperature and pH value were approximately 45 °C and 8.0 respectively. Mn^2+^ was an activator of neutral proteinase. The glucoamylase had the maximum activity at 35 °C, and was activated by Cu^2+^, Fe^3+^ and Mn^2+^. Its optimum temperatures and pH value were 35 °C and 8.0 respectively. The pectinase had the maximum activity at 40 °C, and was activated by Ca^2+^ and Mn^2+^. Its optimum temperatures and pH value were 35–40 °C and 6.0 respectively. The optimum conditions of CCSKO extraction by AEM were also investigated. The results suggested that the best amount of enzyme solution and enzymolysis time were 20% (v/v) and 4 h, respectively. The oil extraction rate was 95.2% under these conditions. Thus, a suitable mutated strain was selected for CCSKO extraction by AEM and the optimum extraction conditions were determined.

## Introduction

*Cinnamomum camphora* (*Lauraceae*) is a plant with high economic values (Babu et al. 2003). The content of medium chain fatty acid triglyceride (MCT) in *Cinnamomum camphora* seed kernel oil (CCSKO) was high (MCT > 94%), and MCT was reported to reduce the consumption and subsequent weight gain of animals (Mumme and Stonehouse 2015; Ferreira et al. 2013; Wang et al. 2016) and improve blood lipid and glucose levels (Page et al. 2009; Fu et al. 2015). There has been a growing interest in CCSKO and its extraction. Meanwhile, MCT was found to have a strong inhibitory effect on Gram-positive bacteria, Gram-negative bacteria, mould and enzymes (Hui et al. 2009; Luo et al. 2014).

In industrial processes, edible oil was obtained by pressing, or a process that combines pressing and organic solvent (Rosenthal et al. 2001). In consideration of environmental protection, aqueous enzymatic method (AEM) has been well accepted by researchers. The energy consumption of edible oil extraction by AEM was low and the process was safe, which was recognized as eco-friendly technology for extraction of bioactive compounds and reflects the development direction for oil research and production (Yusoff et al. 2015; Azmir et al. 2013; Puri et al. 2012). The product yield by AEM may be increased by using enzymes which can hydrolyse the proteins or other structural components of the seeds. Therefore, it is imperative to identify the strains which produce a high number of enzymes, have a high yield of proteinase and very low yield of lipase.

*Bacillus amyloliquefaciens*, which belongs to the genus *Bacillus*, is widely distributed in nature, such as soil and plant roots. It is easy to be cultivated and is safe to human and animals. The original strain NCU116 was identified from the waste residue from CCSKO production. The proteinase activity of NCU116 was much higher than those of *Bacillus* sp. NPST-AK15 (Ibrahim et al. 2015), *Bacillus subtilis* (Pant et al. 2015) and *Bacillus licheniformis* RP1 (Haddar et al. 2011). On the other hand, the lipase activity of NCU116 was very low (Zeng et al. 2017), which makes it a suitable strain for CCSKO extraction by AEM. Although the proteinase secreted by *B. amyloliquefaciens* NCU116 exhibited promising properties, and the proteinase production was increased to 6984.3 U/ml through optimizing cultivation conditions, the yield of proteinase by NCU116 was unsatisfactory for CCSKO extraction by AEM.

Researchers used UV, microwave radiation (Wang et al. 2007; Subhedar and Gogate 2014; Li et al. 2010), combined physical and chemical methods (Afifi et al. 2014; Lian et al. 2010) or site-directed mutagenesis (Jaouadi et al. 2010; Hernandez and Fernandezlafuente 2011) to improve the enzyme performance of original strains. The strains used in the fermentation industry or food industry were improved by mutagenesis instead of genetic modification. Therefore, the key to improving the yield and quality of CCSKO is to screen a strain with high proteinase and low lipase activities and can grow in the MCT-containing medium. In this work, we have screened a new bacterial strain which had a high proteinase activity and can be used for medium-chain triglycerides extraction and the properties of its extracellular enzymes were investigated. The optimum fermentation times of the extracellular enzymes and the optimum oil extraction conditions were also determined.

## Materials and methods

### Bacteria and reagents

The organism was *B. amyloliquefaciens* NCU116 identified by our laboratory (Zeng et al. 2017). Corn flour, bran and soybean meal were purchased from Nanchang Jingke Co. (Nanchang, China). Pectin powder was purchased from Solarbio Science & Technology Co. (Beijing, China). Agar and soluble starch were from Beijing Aobox Biotechnology Co. (Beijing, China). Folin phenol was purchased from Lida Biotechnology Co. (Shanghai, China). Carboxymethyl cellulase sodium was obtained from Sinopharm Chemical Reagent Co. (Shanghai, China). Casein was from Damao Chemical Reagent Co. (Tianjin, China). Na_2_HPO_4_·12H_2_O, KH_2_PO_4_, NaCl, Na_2_CO_3_, NaOH, trichloroacetic acid, and glucose purchased from Xilong Chemical Co. (Guangzhou, China) were of analytical grade.

### Culture media

Bouillon culture medium was comprised of 1% (w/v) peptone, 0.3% (w/v) beef extract and 0.5% (w/v) NaCl, pH 7.0 ± 0.2. Agar culture medium contained 2% (w/v) agar, 1% (w/v) peptone, 0.3% (w/v) beef extract and 0.5% (w/v) NaCl, pH 7.0 ± 0.2. Skim milk powder (1.5%, w/v) was added to the bouillon culture medium to make skim milk bouillon culture medium. Fermentation medium consisted of 4.5% (w/v) corn flour, 2.5% (w/v) wheat bran, 4% (w/v) soya bean meal, 0.2% (w/v) CaCl_2_, 0.03% (w/v) KH_2_PO_4_ and 0.4% (w/v) Na_2_HPO_4_·12H_2_O, pH 7.0 ± 0.2. All these media were sterilized for 20 min at 121 °C in a portable sterilizer.

### UV treatment

The bacterial solution of the strain NCU116 was diluted by normal saline. The bacterial solution (4 ml) was irradiated by UV light for 0.5–30 min (the UV wavelength was 257.3 nm, the power was 15 W, and the distance was 20 cm). After the treatment, the bacterial suspension was coated onto the agar culture medium plate. The plate was covered with a black bag to avoid light damage and cultured overnight at 37 °C, and then the number of bacterial colonies on the plate was recorded. The death rate was calculated using the following formula:

$${\text I\%}\,=\,\left[1-\left(\text W_{\text t}/ \text W_{\text 0}\right)\right]\,*\,100{\%}$$

where I represents the death rate, W_t_ represents the number of colonies in the UV irradiation group, W_0_ represents the number of colonies in the blank group.

### NTG treatment

The bacterial liquid with the death rate of 85% after UV treatment was selected, and coated onto the skim milk bouillon culture medium and incubated at 37 °C for 24 h. The strains above were selected by the ratio of H/C and inoculated into the bouillon culture medium. The medium was incubated at 37 °C, 220 rpm, for 12 h. Then, the agar culture medium was coated with 0.1 ml bacterial suspension. The medium was inoculated with a small amount of NTG powder by a sterile toothpick. NTG inhibition zone was observed after cultivation at 37 °C for 18 h.

The lawn was scraped from the edge of NTG inhibition zone, and inoculated into bouillon culture medium (5 ml) and incubated at 37 °C, 220 rpm, for 4 h. Then, the diluted bacterial liquid was coated onto skim milk bouillon culture medium and incubated at 37 °C for 24 h. The mutated strains were chosen by the values of H/C, and then these strains were screened with shake flask fermentation at 37 °C, 220 rpm, for 44 h. The mutated strain with the highest proteinase activity was selected. The strain was then observed under an optical microscope and scanning electron microscope.

The proteinase activities were measured according to the methods by Pant et al. (2015) and Wang et al. (2007). Enzyme solution (1 ml) was diluted with phosphate buffer (pH 7) and mixed with casein (1%). The mixture was incubated at 40 °C for 10 min. Then 2 ml trichloroacetic acid (0.4 mol/l) were added to the mixture to stop the reaction. The mixture was centrifuged (6640*g*, 10 min) and the supernatant was collected. Then the supernatant (1 ml) was mixed with Na_2_CO_3_ (5 ml) and Folin-phenol reagent (1 ml), and incubated for 20 min at 40 °C. The absorbance at 680 nm was measured in an UV spectrophotometer. The activities were measured by repeating three times. One unit of enzyme activity (U/ml) was defined as 1 ml enzyme hydrolysis of casein to release 1 μg tyrosine per minute under these conditions.

### Effect of fermentation time on the activities of extracellular enzymes

In order to analyze extracellular enzymes activities at a certain fermentation time, the effects of fermentation time on the activities of extracellular enzymes were determined. When the cultivation times were between 38 and 48 h, it was sampled every 2 h. The fermentation solution was centrifuged at 4250*g* for 10 min, the supernatants obtained at different fermentation times contained the fermentation enzyme samples. The activities of proteinase, glucoamylase, pectase, amylase and cellulase were measured at pH 7, 40 °C. Then, the optimum fermentation times for the extracellular enzymes were determined.

Pectinase activity was determined according to QB 1502-92 (1992). One unit of pectinase activity was defined as the amount of enzyme needed to produce 1 mg of galacturonic acid per hour under assay conditions. Glucoamylase activity was determined according to GB 8276-2006 (2006). One unit of glucoamylase activity was defined as the amount of enzyme produced 1 mg of glucose per hour under assay conditions. Cellulase activity was determined using the same method as Berlin et al. (2006) with modifications. One unit of cellulase activity was expressed as 1 μmol of glucose liberated per minute under assay conditions. Amylase activity was determined using the method of GB 8275-2009 (2009). One unit of amylase activity was defined as 1 g soluble starch liquefied per hour under assay conditions.

### Characterization of extracellular enzymes

The activities of extracellular enzymes were measured in different pH values (4–10) and at different temperatures (35–65 °C). The thermal stability was measured at different incubation times (30, 60, 90 and 120 min). In addition, the activities of the proteinase in the presence of 0.01 mol/ml metal ions (Mn^2+^, Mg^2+^, Ca^2+^, Cu^2+^, Zn^2+^, Fe^2+^ and Fe^3+^) were measured.

### Extraction of CCSKO by AEM

To determine the efficiency of AEM by using the strain NCU116-1, the conditions of AEM were studied. The effect of enzymolysis times (1–5 h) on oil extraction rate was determined. To study the optimum amount of enzyme solution, the addition amounts were adjusted to 5% (v/v), 10% (v/v), 15% (v/v), 20% (v/v) and 25% (v/v), respectively. The extraction of CCSKO by AEM was conducted using our method (Zeng et al. 2015).

### Data analysis

Results were expressed as the mean ± standard deviation (SD). Data were analysed using one way analysis of variance (ANOVA), followed by independent-sample *t* test (Statistics programming software SPSS 19.0, Chicago, USA.). A *p* value <0.05 was considered to be statistically significant.

## Results

### Mutagenesis

The results of UV mutation are shown in Table 1. The death rate of the strain NCU116 increased rapidly from 0 to 2 min. When ultraviolet irradiation time was 3 min, the death rate was 95.7% and only few strains survived. In order to obtain high variability, 80–90% death rates were chosen as a condition for mutagenesis. Therefore, the optimal mutation time was 2 min.

**Table 1:** The results of UV mutation

Irradiation time (min)	Number of colonies	Death rate (%)
0	162	0
0.5	120	25.9
1	64	60.5
2	19	88.3
3	7	95.7

Thirteen strains with the values of H/C greater than 3.4 were picked from the skim milk bouillon culture medium after NTG treatment. As shown in Table 2, the proteinase activity (9116.1 ± 58.2 U/ml) of the strain Y6 was the highest among them, with an increase of 31.9% compared with the original strain. Moreover, the lipase activity of Y6 was not detected. The strain Y6 was thus selected for the following study and was designated as *B. amyloliquefaciens* NCU116-1.

**Table 2:** Results of compound mutation

Strain number	H/C	Proteinase activity (U/ml)	Strain number	H/C	Proteinase activity (U/ml)
NCU116	3.4	6912.3 ± 23.1^a^	Y7	3.9	7124.0 ± 25.1^d,h^
Y1	3.6	6880.1 ± 32.2^a^	Y8	5.0	6099.5 ± 10.2^g^
Y2	3.9	7533.0 ± 20.7^b^	Y9	5.1	7441.2 ± 63.6^b^
Y3	3.1	4019.4 ± 12.0^c^	Y10	4.1	7086.8 ± 53.5^h^
Y4	4.6	7199.8 ± 49.2^d^	Y11	4.4	3321.7 ± 21.2^i^
Y5	4.8	7948.8 ± 35.6^e^	Y12	5.5	7914.0 ± 13.5^e^
Y6	5.3	9116.1 ± 58.2^f^	Y13	3.6	7019.8 ± 18.0^h^

Y1–Y13 indicates that different strains obtained by mutagenesis. Values represent the mean ± SD of three replicates. The different letters mean significant differences. ^a–i^ Significant difference at *p* < 0.05

**Fig. 1:**
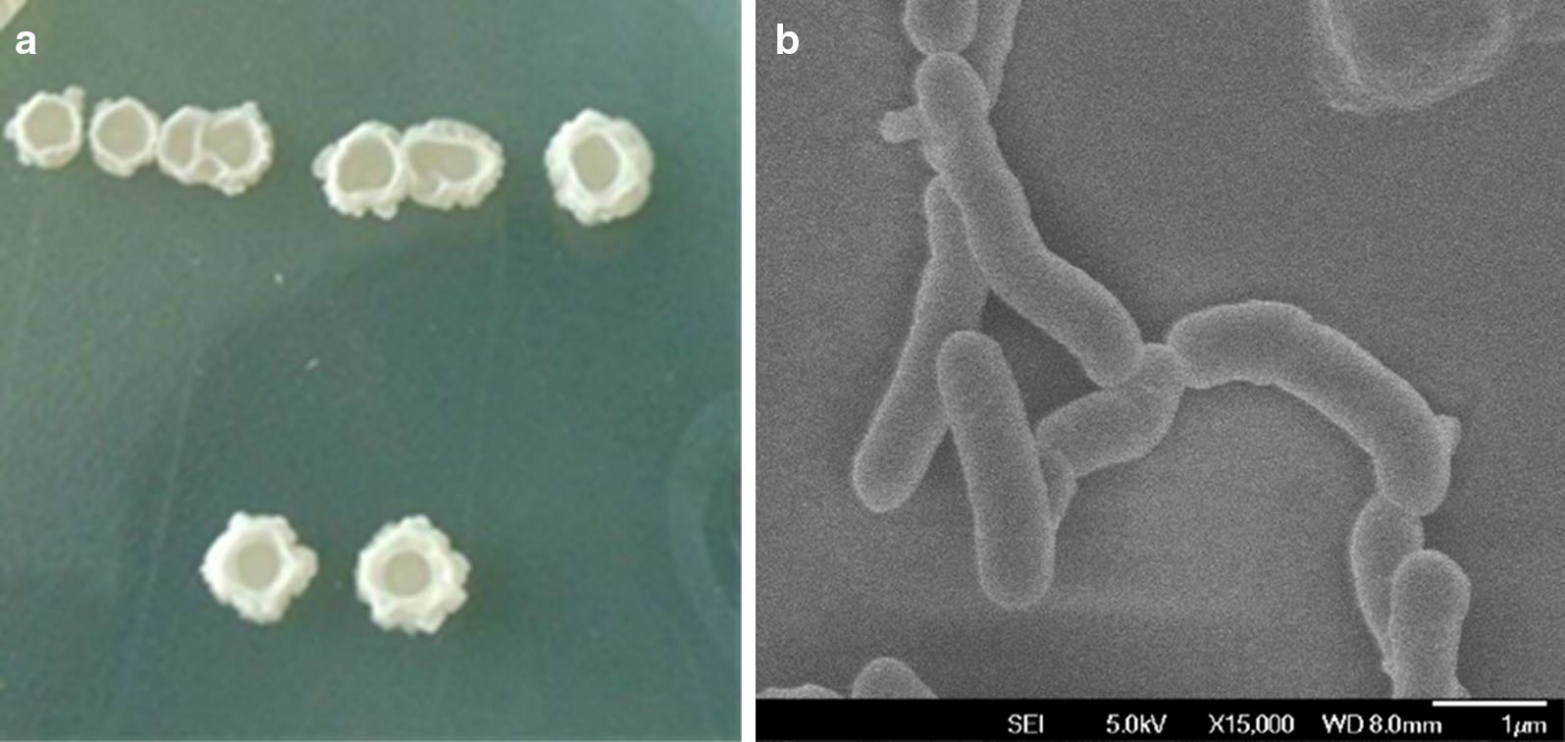
Colony morphology of strain NCU116-1 (**a**) and its form observed by SEM (**b**). Magnification of SEM is ×15,000

The strain grew rapidly when cultured in nutrient agar plate. As shown in Fig. 1a, the colonies of NCU116-1 were round, the size was 5.0–8.0 mm and the surface was smooth with circular protrusions. The appearance of the strain observed by a scanning electron microscope (SEM) is shown in Fig. 1b. The strain was rod-shaped with slightly bent, its surface was smooth, the length was 1.5–2.5 µm, and the diameter was 0.4–0.5 µm.

### Determination of optimum fermentation time

**Fig. 2:**
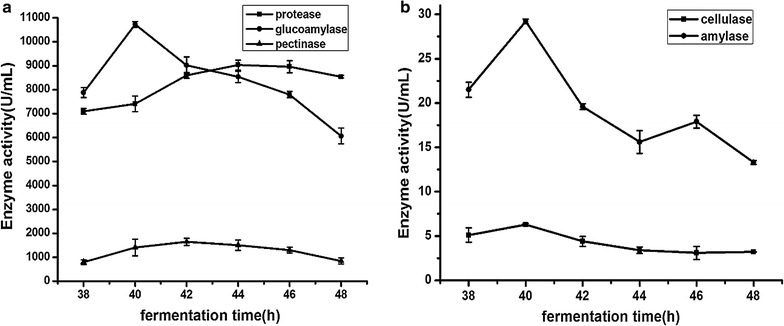
The relationship between fermentation time and exoenzyme activity in **a** and **b**. The proteinase, glucoamylase and pectase are shown in **a**. Cellulase and amylase are shown in **b**. The activities of enzymes were sampled every 2 h when the cultivation time was between 38 and 48 h. Data are mean ± SD (n = 3). The types and optimum fermentation times of the extracellular enzymes were determined

As shown in Fig. 2, extracellular enzymes of the strain NCU116-1 included proteinase, glucoamylase, pectase, cellulase and amylase. The activity of proteinase was the highest (9027.0 U/ml) with shaking flash fermentation for 44 h. The activities of glucoamylase, cellulase and amylase were the highest (10727.6, 6.3 and 29.2 U/ml, respectively) with shaking flash fermentation for 40 h. The activity of pectase was the highest (1644.2 U/ml) with shaking flash fermentation for 42 h. Then, the optimum fermentation time for the extracellular enzymes was determined. When the fermentation time was 44 h, each exoenzyme secreted by the strain NCU116-1 remained at a high level, especially the activity of proteinase. Consequently, the fermentation supernatant was chosen for CCSKO extraction by AEM when the fermentation time was 44 h.

### Properties of the extracellular enzymes

#### Proteinase

**Fig. 3:**
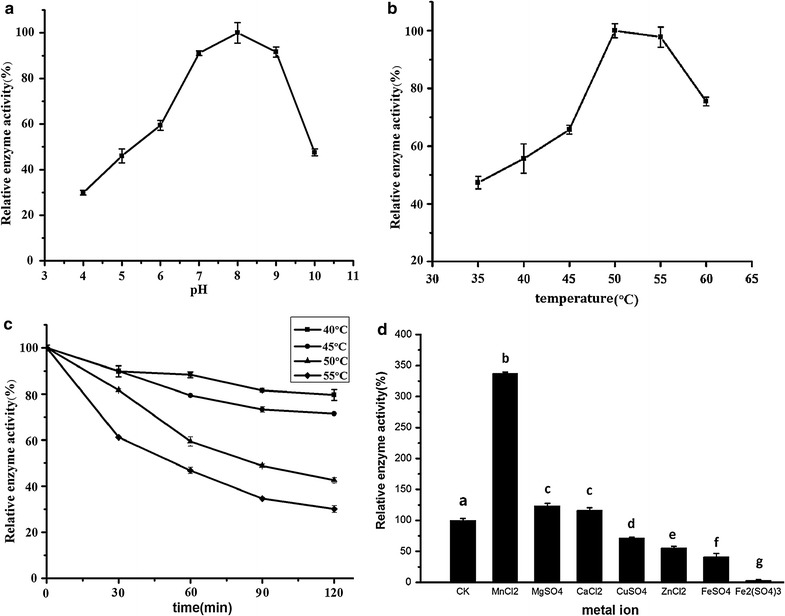
The effects of pH (**a**), temperature (**b**), incubation time (**c**) and metal ion (**d**) on the activities of proteinase. The pH optimum of proteinase was studied from pH 4–10. The temperature optimum was studied at 35–60 °C. Temperature stability was determined by holding the enzyme at 40–55 °C for 120 min. Metal ions included Mn^2+^, Mg^2+^, Ca^2+^, Cu^2+^, Zn^2+^, Fe^2+^ and Fe^3^. Values represent the mean ± SD of three replicates. Values with different letters indicate significant differences (*p* < 0.05)

The effects of pH, temperature, incubation time and metal ion on the activity of proteinase are shown in Fig. 3. The enzyme exhibited high activities at alkaline pH (in the range of pH 7.0–9.0), and the activity was the highest at pH 8.0 (Fig. 1a). As shown in Fig. 1b, the activity of proteinase was the highest at 50 °C. The activity of proteinase was stable for 120 min at 40 and 45 °C (Fig. 1c). When the temperatures reached 50 °C or higher, the activity of proteinase decreased rapidly with an increase in incubation time. Thus, the most suitable temperature for proteinase was approximately 45 °C. Mn^2+^ was found to improve proteinase activity significantly (Fig. 1d), suggesting that the proteinase produced was metalloproteinase. Compared with the original strain NCU116, the proteinase activity of the mutated strain increased 31.9%. Meanwhile, the most suitable pH changed from 7 to 8, and the most suitable temperature changed from 40 to 45 °C. The main components of the camphor tree seed kernel are oil and proteins. Proteins in seed kernel can be hydrolyzed by proteinase during CCSKO extraction by AME. The study on proteinase properties can help us determine the most suitable conditions of AME.

#### Glucoamylase

**Fig. 4:**
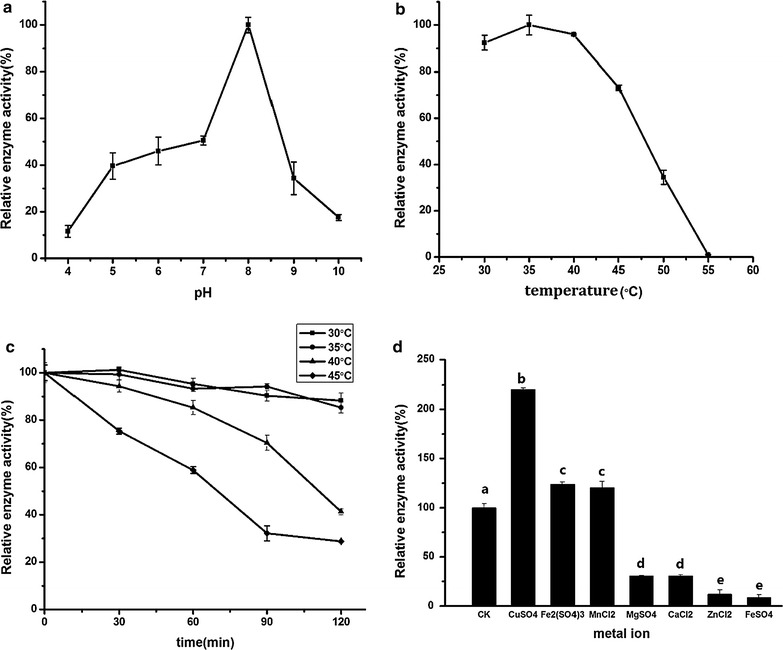
The effects of pH value (**a**), temperature (**b**), incubation time (**c**) and metal ion (**d**) on the activity of glucoamylase. The pH optimum of proteinase was studied from 3 to 11. The temperature optimum was studied from 30 to 60 °C. Temperature stability was determined by holding the enzyme at 40–55 °C for 120 min. Metal ions included Mn^2+^, Mg^2+^, Ca^2+^, Cu^2+^, Zn^2+^, Fe^2+^ and Fe^3+^. Values represent the mean ± SD of three replicates. Values with different letters indicate significant differences (*p* < 0.05)

The effects of pH value, temperature, incubation time and metal ion on the activity of glucoamylase produced by NCU116-1 are shown in Fig. 4. The glucoamylase was active over a range of pH values (4–10), with the maximum activity at pH 8 (Fig. 4a). Glucoamylase’s suitable pH values were 7–8. These results showed that glucoamylase had a high activity under neutral conditions which was suitable environment for aqueous enzymatic extraction. As shown in Fig. 4b, the activity of glucoamylase was the highest at 35 °C. The activity of glucoamylase was stable for 120 min at 30 and 35 °C (Fig. 4c). When temperature reached 40 °C and higher, the activity of glucoamylase decreased rapidly as incubation time increased. Thus, the most suitable temperature for glucoamylase was 30–35 °C. As shown in Fig. 4d, Cu^2+^, Fe^3+^ or Mn^2+^ improved the activity of glucoamylase. It was found that the activity of glucoamylase was significantly inhibited by Mg^2+^, Fe^2+^, Zn^2+^ and Cu^2+^. The results showed that Cu^2+^ was the main effective activator of glucoamylase as it was able to stimulate the activity of glucoamylase about 145%. Thus, glucoamylase may play a role in aqueous enzymatic extraction of CCSKO.

#### Pectinase

**Fig. 5:**
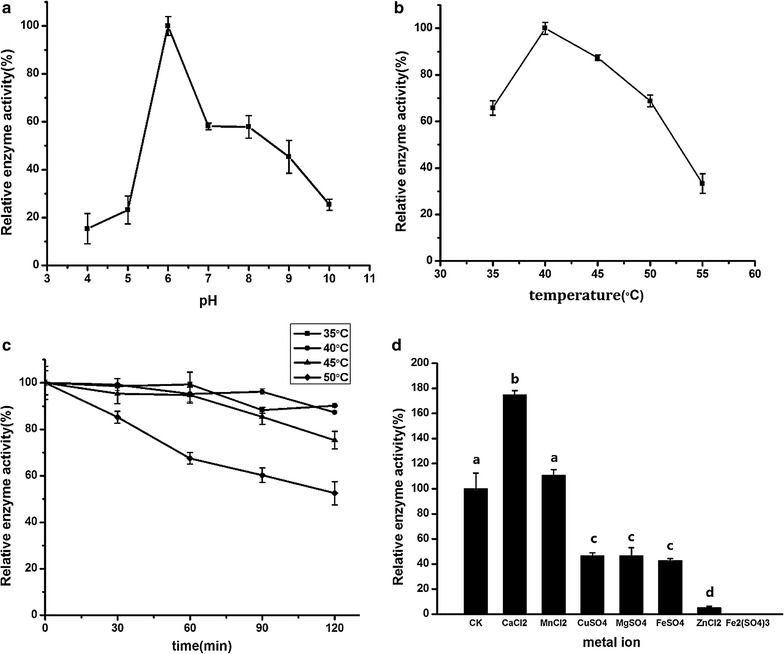
The effects of pH value (**a**), temperature (**b**), incubation time (**c**) and metal ion (**d**) on the activity of pectinase. The pH optimum of proteinase was studied from 3 to 11. The temperature optimum was studied from 35 to 60 °C. Temperature stability was determined by holding the enzyme at 35–50 °C for 120 min. Metal ions included Mn^2+^, Mg^2+^, Ca^2+^, Cu^2+^, Zn^2+^, Fe^2+^ and Fe^3+^. Values represent the mean ± SD of three replicates. Values with different letters indicate significant differences (*p* < 0.05)

The effects of pH value, temperature, incubation time and metal ion on the activity of pectinase produced by NCU116-1 are shown in Fig. 5. The activity of pectinase was the highest when pH was 6 (Fig. 5a). Pectinase’s suitable pH values were 6–8. The activity of pectinase was the highest at 40 °C (Fig. 5b). As shown in Fig. 5c, the activity of pectinase was stable for 120 min at 35 and 40 °C. When temperature reached 45 °C and higher, the activity decreased with an increase in incubation time. Thus, the most suitable temperatures for pectinase were 35–40 °C. The results showed that Mn^2+^ or Ca^2+^ improved the activity of pectinase, which was able to stimulate the activity of pectinase about 75 and 10% (Fig. 5d). Pectin may have the function of emulsification to the oil, and it could be hydrolysed by pectinase to β-galacturonic acid. Pectinase could also play an assistant role in the process of aqueous enzymatic method.

### Condition optimization of AEM

**Fig. 6:**
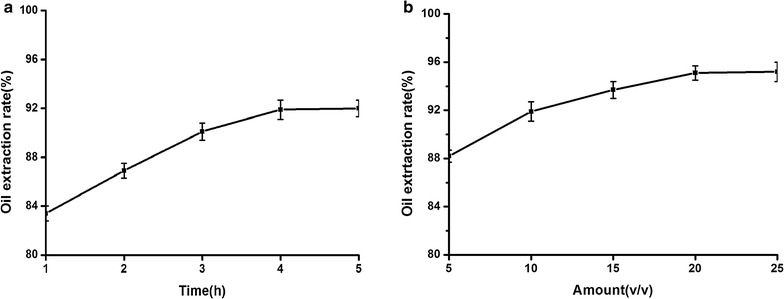
The effect of enzymolysis time (**a**) and amount of enzyme solution (**b**) on oil extraction rate. Enzymolysis times were 1, 2, 3, 4 and 5 h, respectively to analyze the effect of enzymolysis times on oil extraction rate. To study the optimum amount of enzyme solution, the addition amounts were adjusted to 5% (v/v), 10% (v/v), 15% (v/v), 20% (v/v) and 25% (v/v), respectively. Data are mean ± SD (n = 3)

Enzymolysis time and amount of enzyme solution were the two crucial factors for the extraction of CCSKO by AEM. As shown in Fig. 6a, b, with the increase of enzyme solution and enzyme hydrolysis time, CCSKO yield gradually increased. But when the amount of enzyme solution was more than 20% (v/v) or the hydrolysis time was longer than 4 h, the oil yield almost no longer increased. This may be because the proteins have been broken down by proteinases. Thus, the best amount of enzyme solution and enzymolysis time were 20% (v/v) and 4 h, respectively. The CCSKO extraction rate was 95.2% under these conditions, which was increased by 4.1% compared with the original strain and reflected high efficiency of oil extraction (Zeng et al. 2015).

## Discussion

Appearance and morphology of the colony in mutated strain under SEM had no significant difference from the original strain, but the extracellular enzyme activity changed greatly. The results may be due to the effects of physical and chemical mutagenesis on the original strain, the DNA which controlled extracellular traits has been mutated as well, but there was no significant change in the colony and cell morphology. Compared with the original strain, the activities of protease, pectinase, glucoamylase and amylase were increased by 107.2, 122.1, 163.5 and 23.8%, respectively. However, the activity of cellulase was reduced by approximately 49%. This may be due to the strong random mutation induced by physical and chemical mutagenesis, the negative mutation happened in cellulase. In some cases, mutagenesis can’t improve the enzyme activity of the strain, and the results have some randomness and contingency.

The activities of proteinase, pectase, glucoamylase and amylase in the mutated strain NCU116-1 increased to varying degrees compared with the original strain NCU116, but the cellulase activity decreased. The properties of proteinase changed as well. Afifi et al. (2014) used gamma radiation and ethyl methanesulfonate to obtain a mutated strain EMS-1, and found that the mutant produced a higher alkaline proteinase activity than the wild strain. Lian et al. (2010) used UV and NTG mutation methods to screen a mutant strain which improved docosahexaenoic acid production than the original strain. The strain’s DNA molecules can form two pyrimidine dimers due to UV irradiation, cause double chain structure distortion and hinder the normal pairing between the bases, leading to the bacterial mutation. NTG is a very effective mutagen, and may change GC into AT in the DNA chain. The combination of these two effects induced a series of variations in the strains.

Camphor tree seed kernel mainly contains proteins (18%) and oil (40%), with some other minor components including cellulose, pectin and amylum. The proteins which form camphor tree seed kernel cells and lipid body membranes may be hydrolyzed by proteinases and the oil released (Rosenthal et al. 2001). The yield of CCSKO extraction by AEM may be improved when the strain can produce a higher proteinase activity. Therefore, the key of the research is to improve the strain’s proteinase activity and determine its properties. Meanwhile, the oil can be adsorbed by the cellulose, and pectin had the function of emulsification to the oil (Funami et al. 2007). Therefore, they may affect the efficiency of AEM. On the other hand, amylum may be hydrolyzed to maltose by amylase, and maltose may be further hydrolyzed to glucose by glucoamylase. Thus, pectase, glucoamylase, cellulase and amylase secreted by the strain NCU116-1 play important roles in AEM.

In summary, the proteinase activity of the original strain was enhanced by a combination of UV with NTG mutation. The mutated strain NCU116-1 exhibited a higher proteinase activity than the original strain and good genetic stability. The extracellular enzymes of NCU116-1 included proteinase, glucoamylase, pectase, cellulase and amylase. The neutral proteinase had the maximum activity at 50 °C, but was unstable. Its optimum temperature and pH value were approximately 40 °C and 8.0, respectively. Mn^2+^ was the activator of neutral proteinase. Glucoamylase had the maximum activity at 35 °C, and was activated by Cu^2+^, Fe^3+^ or Mn^2+^. Its optimum temperatures and pH value were 30–35 °C and 6.0, respectively. Pectinase had the maximum activity at 40 °C and was activated by Mn^2+^ or Ca^2+^. Its optimum temperatures and pH value were 35–40 °C and 6.0, respectively. The best amount of enzyme solution and enzymolysis time were 20% (v/v) and 4 h, respectively. The oil extraction rate was 95.2% under these conditions which was increased by 4.1% compared with the original strain. Thus, our findings have provided useful information for CCSKO extraction by AEM or other biotechnological applications.

## Abbreviations

AEM: aqueous enzymatic method
CCSKO: *Cinnamomum camphora* seed kernel oil
H/C: the ratio of hydrolytic zone diameter to colony diameter
MCT: medium chain fatty acid triglyceride
NTG: *N*-methyl-*N′*-nitro-*N*-nitroso guanidine
SEM: scanning electron microscope
UV: ultraviolet
